# Assembling single gold nanorods into large-scale highly aligned nanoarrays via vacuum-enhanced capillarity

**DOI:** 10.1186/1556-276X-9-556

**Published:** 2014-10-07

**Authors:** Jiaojiao Wang, Min Li, Bochong Tang, Peng Xie, Lei Ma, Zhongbo Hu, Yuliang Zhao, Zhongqing Wei

**Affiliations:** 1 College of Materials Science and Opto-electronic Technology, University of Chinese Academy of Sciences, Yuquan Rd. 19A, Beijing 100049, China; 2 CAS Key Laboratory for Biomedical Effects of Nanomaterials and Nanosafety, Institute of High Energy Physics, Chinese Academy of Sciences, Yuquan Rd. 19B, Beijing 100049, China

**Keywords:** Gold, Nanorods, Assembly, Capillary, Atomic force microscopy, Nanoarray

## Abstract

We report a simple, straightforward, and efficient approach to assemble single gold nanorods (AuNRs) into highly aligned arrays, via a unique vacuum-enhanced capillarity. The assembled AuNR arrays demonstrate both an excellently unidirectional ordering and a wonderful single-rod resolution. The key role of vacuum in this approach enables high-aspect-ratio (10 to 22) AuNR alignment and efficiently facilitates large-area alignment. Further investigation of one- and two-dimensional AuNR arrays would undoubtedly be beneficial to their potential applications.

## Background

Controlled assembly and alignment of one-dimensional (1D) nanostructures such as nanorods, nanotubes, and nanowires are essential for their integration and applications in many macroscopic devices for nanoelectronics, [1] sensing, [2-4], and plasmonics [5,6]. Although 1D nanostructures such as gold nanoarrays can be fabricated by conventional top-down lithography (e.g., electron beam lithography), the process is time-consuming and the gold involved is polycrystalline, which may ultimately degrade the performance of devices. Recent wet chemical approaches have made it possible to synthesize various anisotropic gold nanostructures including dimeric gold nanorod (AuNR) junctions [4,5,7]. Meanwhile, several novel bottom-up strategies, such as capillary-driven assembly, [8-10] chemically template-directed assembly [11-13], spontaneous self-assembly [14], surface amidation assembly [15], and polymer-based assembly [16-18], have been developed to assemble spherical gold colloid nanoparticles [7] and anisotropic pentahedrally twinned AuNRs into ordered gold nanostructures [11,15,19]. However, the manipulation of anisotropic AuNRs to form highly aligned and ordered nanoarrays still remains significantly challenging. Herein, we employ a strong capillarity enhanced by a vacuum created from a syringe to assemble AuNRs into nanoarray structures. The assembled linear nanoarrays are not only single-rod resolved but also extremely well aligned. Compared with the spontaneous capillary assembly, this approach is simple, quick, and clean (does not involve any other chemicals such as polymers) and can be highly efficient in large-area assembly of AuNRs with high aspect ratios (ARs) (approximately 10 to 22). The vacuum-enhanced capillary force effectively promotes the formation of highly aligned AuNR arrays. This work is an extended study of our previous work on gold nanorods [20].

## Methods

**Figure 1:**
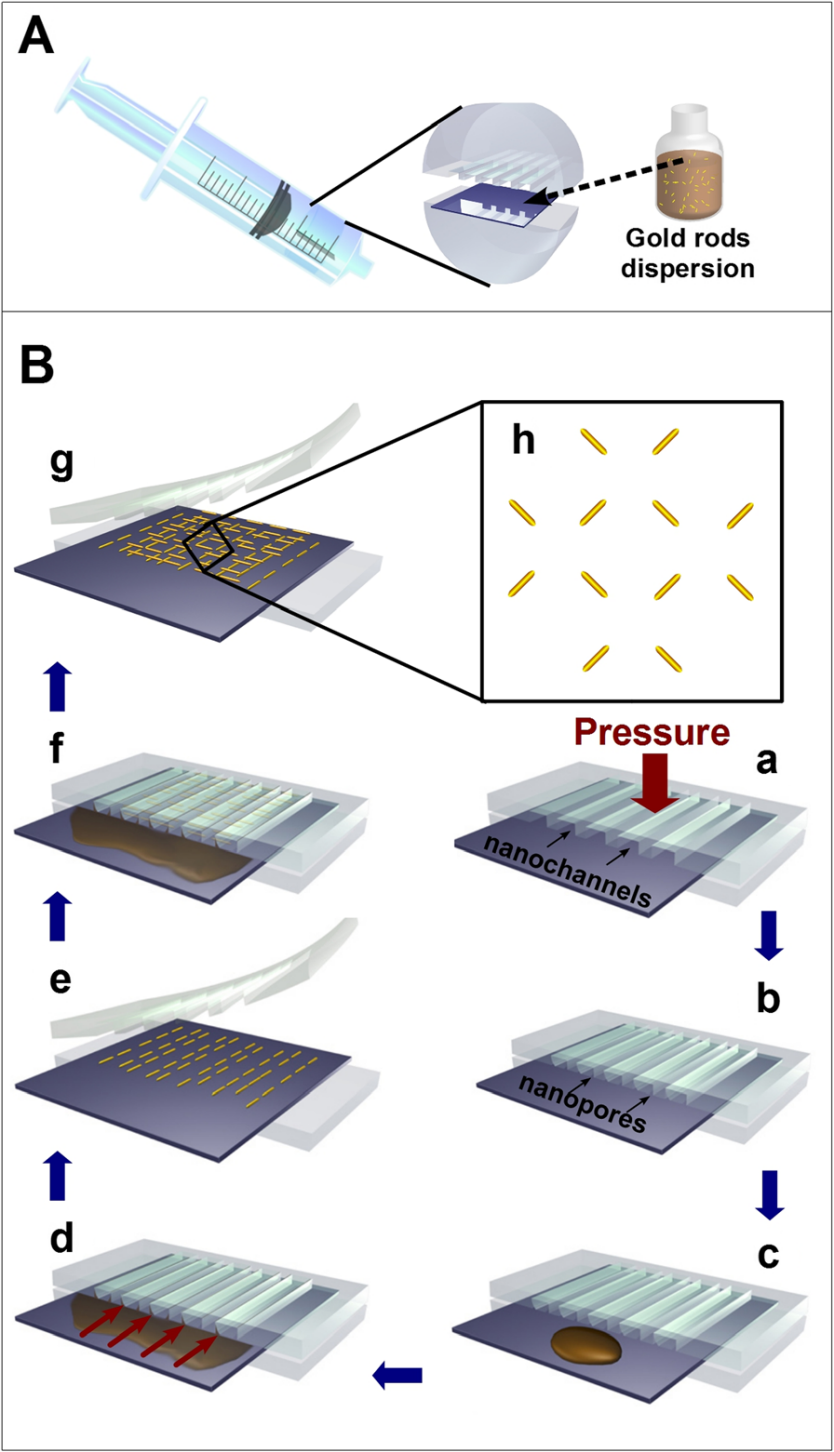
**Schematic drawing depicting the formation of AuNR arrays driven by a vacuum-enhanced capillary force (A, B).** See text for details.

Figure 1 outlines our procedure depicting the assembly of AuNRs using a syringe as a simple pump to generate a vacuum-enhanced capillary force. Briefly, a piece of cleaned Si (100) substrate was sandwiched in between two pieces of semi-cylindrical poly(dimethylsiloxane) (PDMS) stamps, one of which has linear periodic nanochannels (a) molded from a DVD template. Note that in the present work, we used two complementary DVD templates (referred to as template 1 and template 2 hereafter, see Additional file 1: Figure S1 and Additional file 1: Figure S2 for details) to fabricate two types of PDMS stamps with nanochannels. A piece of tape was then wound tightly around the two pieces of PDMS stamps to clamp them together and hold the silicon substrate in position. Following dropping a few drops (approximately 5 μl) of highly purified AuNR dispersion (approximately 0.05 mg/ml) on the protruding portion of the substrate surface, the entire assembly was then carefully squeezed into the cylindrical tube of a syringe after the plunger had been pulled away from the syringe body. At this time, the nanochannels may deform due to the pressure imposed by the internal wall of the tube, giving rise to the formation of nanopore canals (b) with a diameter of ca. 20 nm. The plunger was pushed back into the tube, followed by sealing the open end of the syringe with a piece of tape. Thus, a closed system was created. Pulling the plunger produced a vacuum inside the tube between the ends of the plunger and stamps. Due to the vacuum-enhanced pressure difference between both ends of the stamps along inside the tube, the flow of the AuNR dispersion occurred spontaneously and rapidly to fill up (d) the nanopore canals. After the whole assembly was then left for several hours for the water to evaporate completely, the PDMS stamps were gently removed, leaving patterned AuNR arrays on the silicon surface (e). A crossed AuNR array can be formed by repeating steps from (a) to (e) to generate another linear array perpendicular to the existing linear array (f-h).

## Results and discussion

**Figure 2:**
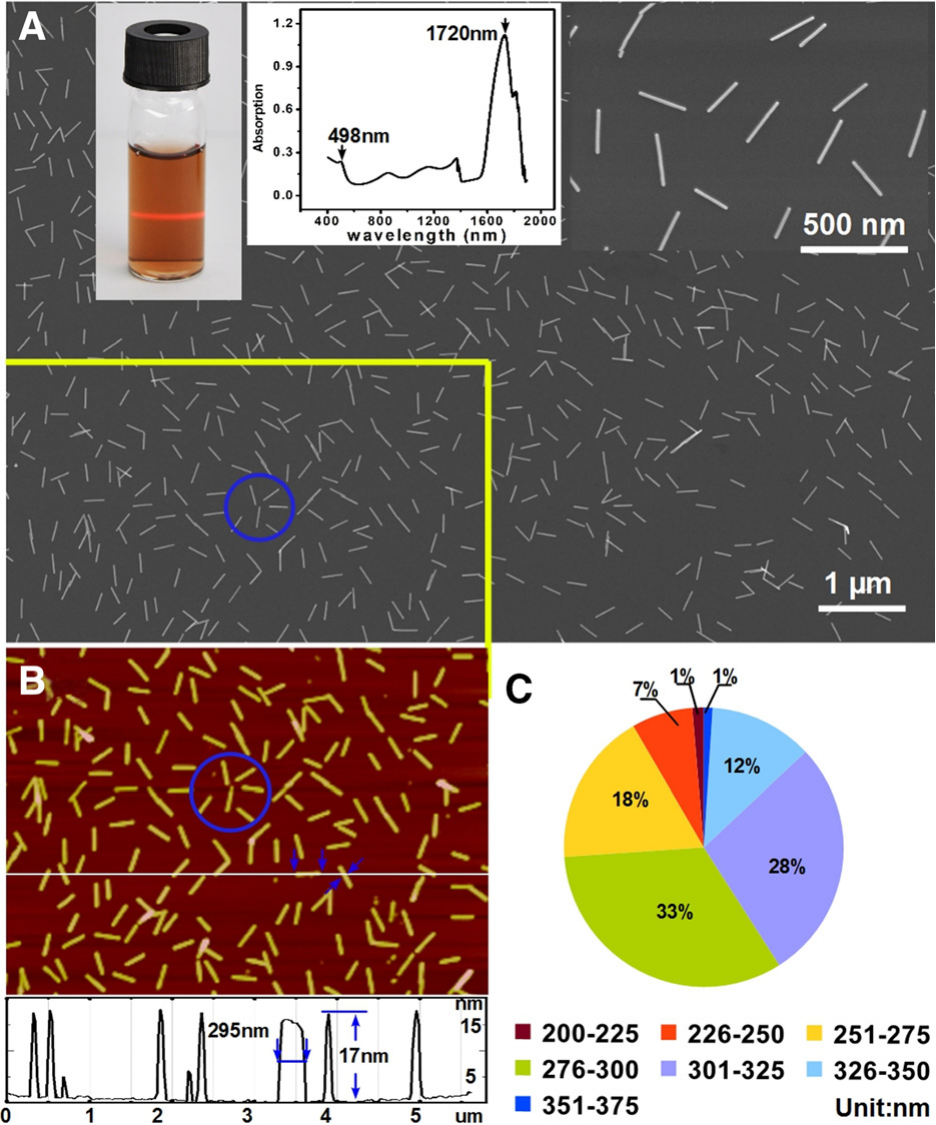
**Highly purified AuNRs used to fabricate AuNR arrays. (A)** SEM image of AuNRs uniformly distributed on a MPTMS-modified silicon surface. Insets are a digital picture of AuNR dispersion (approximately 0.1 mg/ml) showing the Tyndall effect, UV-Vis spectrum, and close-up view of AuNRs, respectively. **(B)***In situ* AFM image of AuNRs captured in the same area as outlined area in the SEM image. The circled items in the two images are used as markers to demonstrate the same areas. **(C)** Statistical distribution of lengths of 722 AuNRs.

We first synthesized AuNRs following the procedure described by Murphy et al. [21] and then purified them following the procedure reported by Zubarev and Khanal [22] [see Additional file 1: Figure S3]. In Figure 2, the aqueous dispersion of thus-prepared AuNRs shows the Tyndall effect, revealing its colloidal nature. Typically, its UV-Vis-NIR spectrum shows one transversal surface plasma peak at about 498 nm and one longitudinal peak at about 1,720 nm. The redshifted maximum of the longitudinal plasmon absorption and the blueshifted maximum of the transverse plasmon absorption are due to the high AR (ca. 10 to 22, see below) of the AuNRs. Figure 2A shows a representative SEM image of AuNRs distributed on the surface of the n-type Si (100) substrate which was previously functionalized with (3-mercaptopropyl)triethoxysilane (MPTES) [see Additional file 1]. Obviously, the AuNRs are highly purified (approximately 100%), size uniform, and well isolated (no aggregates). Larger-area observations (>55 μm, Additional file 1: Figure S4) also show the same situation. We measured the size of 722 single AuNRs (not all of them are shown) in the AFM image (Figure 2B), which was captured in the same area as outlined in the SEM image (Figure 2A). AFM measurements indicate that all of the AuNRs possess a length of over 200 nm with a diameter of ca. 17 to 20 nm. Shown in Figure 2C is a pie chart depicting the statistics analysis of lengths for measured AuNRs. The average high AR for AuNRs ranges from ca. 10 to 22.

**Figure 3:**
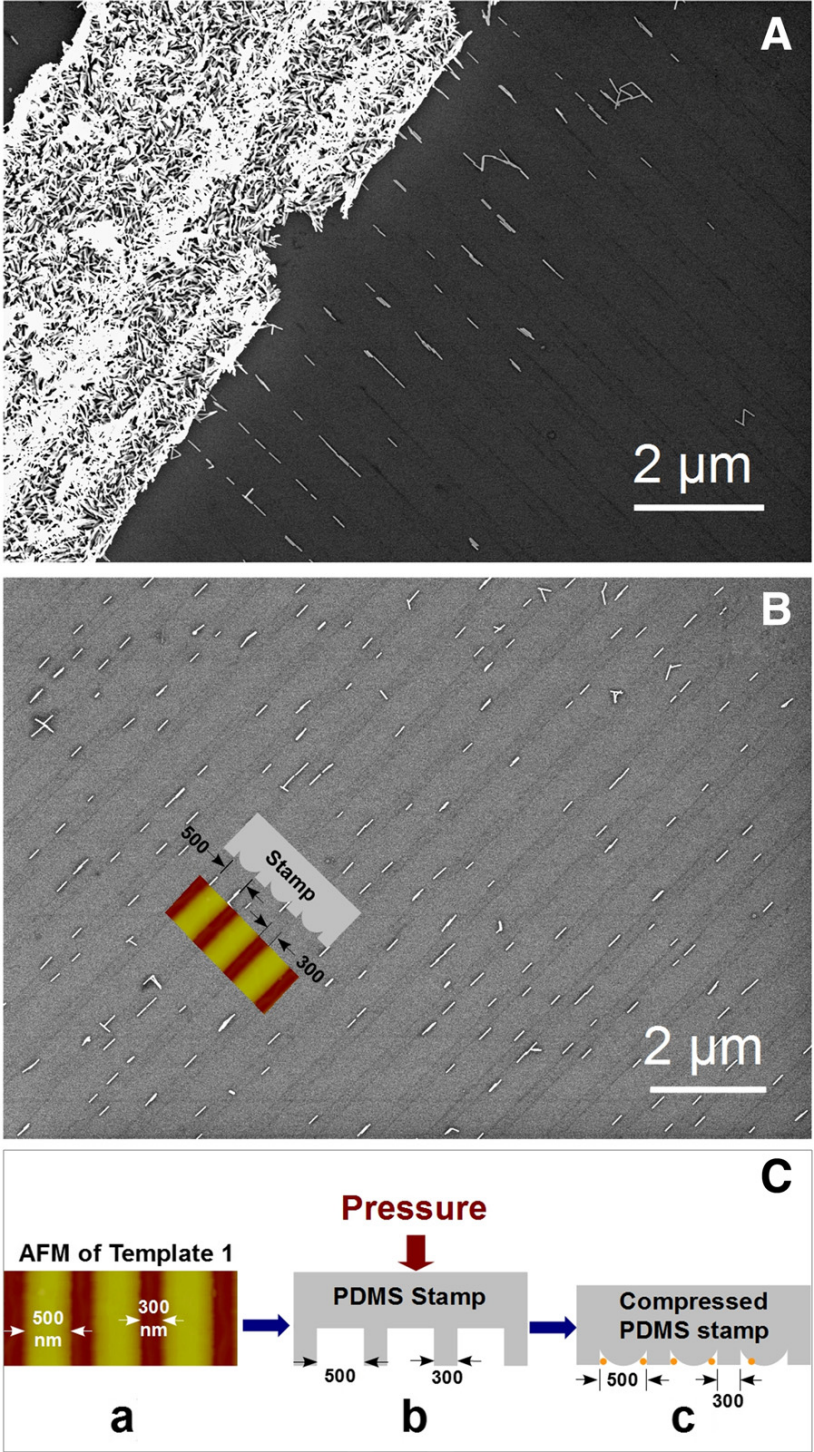
**Highly aligned AuNRs generated via the procedure described in Figure **1**. (A)** Aggregated and single AuNRs before and after entering nanopores, respectively. **(B)** Representative AuNR array showing a resolution of single AuNRs. **(C)** Model used to illustrate the process of array formation.

The SEM images presented in Figure 3 show highly aligned AuNR arrays assembled on silicon surfaces with the aid of a vacuum-enhanced capillary force. It is obvious that the AuNRs were aligned extremely well (Figure 3B). The spacing in the periodic array structure is determined exactly by the dimension of the PDMS stamp used. In Figure 3A, while some AuNRs succeeded in entering nanopores under vacuum-enhanced capillary and then aligning, most of the single AuNRs accumulated vastly on the entry positions, giving rise to AuNR aggregations. The schematic diagram shown in Figure 3C is the mechanism we proposed to illustrate the entire process of forming aligned arrays. Under the pressure imposed by the internal wall of the syringe tube, the PDMS stamp (b), molded from template 1 (a), sagged down to generate nanopore canals. The size of the resulting canals matched the diameter (approximately 20 nm) of AuNRs so well that only one single AuNR could enter the canal at a time, leading to a highly aligned linear array at a single-AuNR level. Large-area aligned AuNR arrays can be seen in Additional file 1: Figure S5. The complete match in Figure 3B between the model (inset) and the alternatively bright and dark press marks left by the PDMS stamps strongly supports our proposed mechanism.

**Figure 4:**
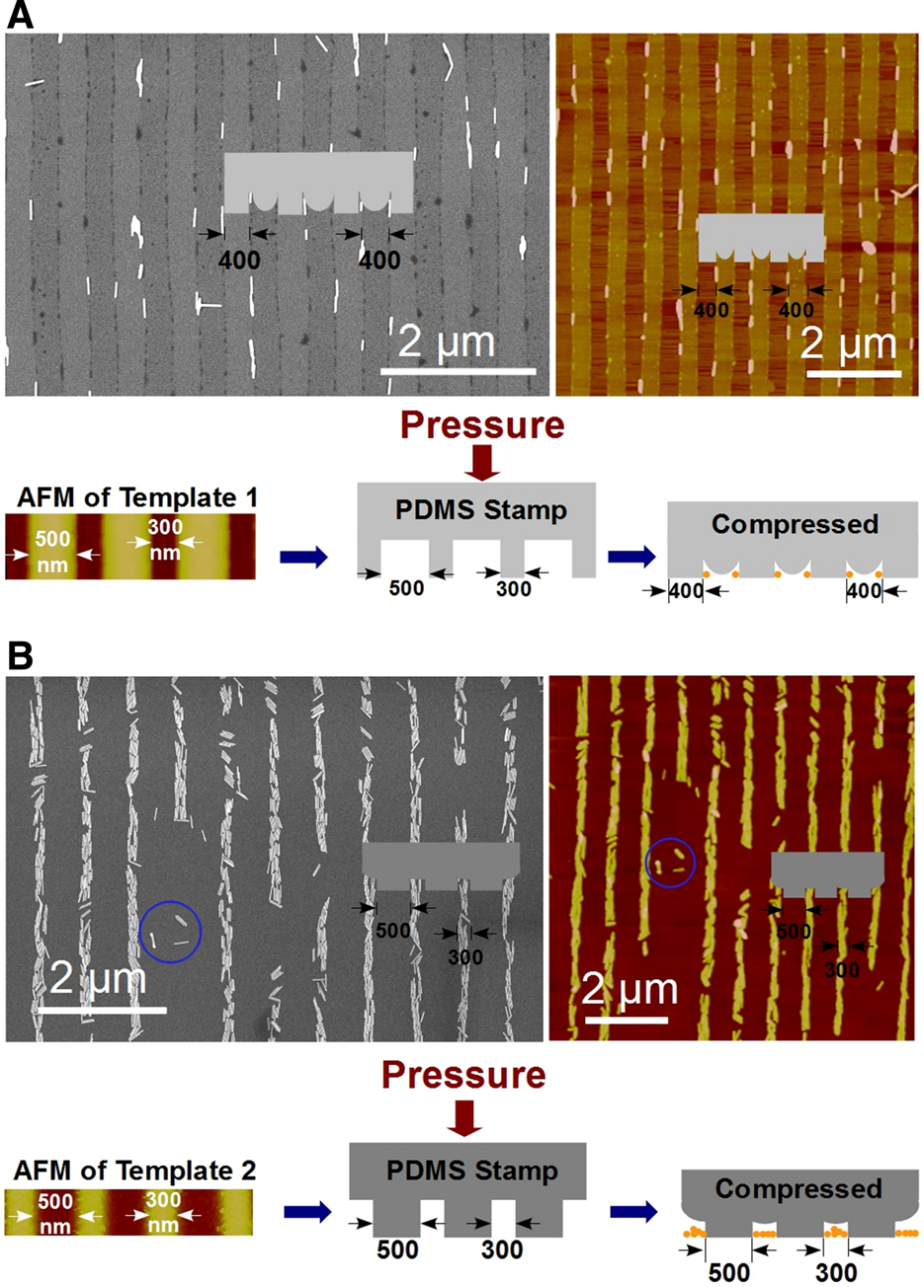
**The structure of AuNR arrays can be tuned by adjusting parameters related to PDMS stamps. (A)** SEM and AFM images of an equally spaced array assembled using a relatively soft stamp. Shown below the images is a model to illustrate the process. **(B)** SEM and corresponding *in situ* AFM images of an array (composed of bundles of AuNRs) assembled by applying smaller pressure to the stamp. The blue circled items in the two images are used as markers to demonstrate the same areas. Shown below the images is a model to illustrate the process.

Subtle differences (e.g., the elastic modulus of stamps, the pressure on stamps) in practical operations can result in different patterning. Shown in Figure 4 are two variants of the AuNR arrays presented in Figure 3. In Figure 4A, a relatively soft PDMS stamp cast from template 1 [see Additional file 1] was used to assemble AuNRs. The array generated has an equally spaced array structure (a period of approximately 400 nm). We speculate that when the soft stamp is in contact with the silicon surface, the raised areas of the stamp deform laterally as a result of the pressure, causing the size increase laterally (from approximately 300 to 400 nm) in contact areas and size decrease laterally (from approximately 500 to 400 nm) in noncontact areas (channel areas). The schematic drawing below the images depicts this process of size changes. Thus, by tuning the elastic modulus of the stamp or/and the pressure imposed on the stamp, linear AuNR arrays with varied spacing can be obtained. Another example shown in Figure 4B demonstrates the effect of less pressure applied to the stamp on the final patterning. In Figure 4B, the stamp (molded from template 2) exerted less pressure and the nanochannels failed to turn into nanopores. As a result, bundles of AuNRs, rather than single ones, entered the nanochannels, giving rise to an array composed of bundles of AuNRs. An additional image of the bundled AuNR array can be seen in Additional file 1: Figure S6.

**Figure 5:**
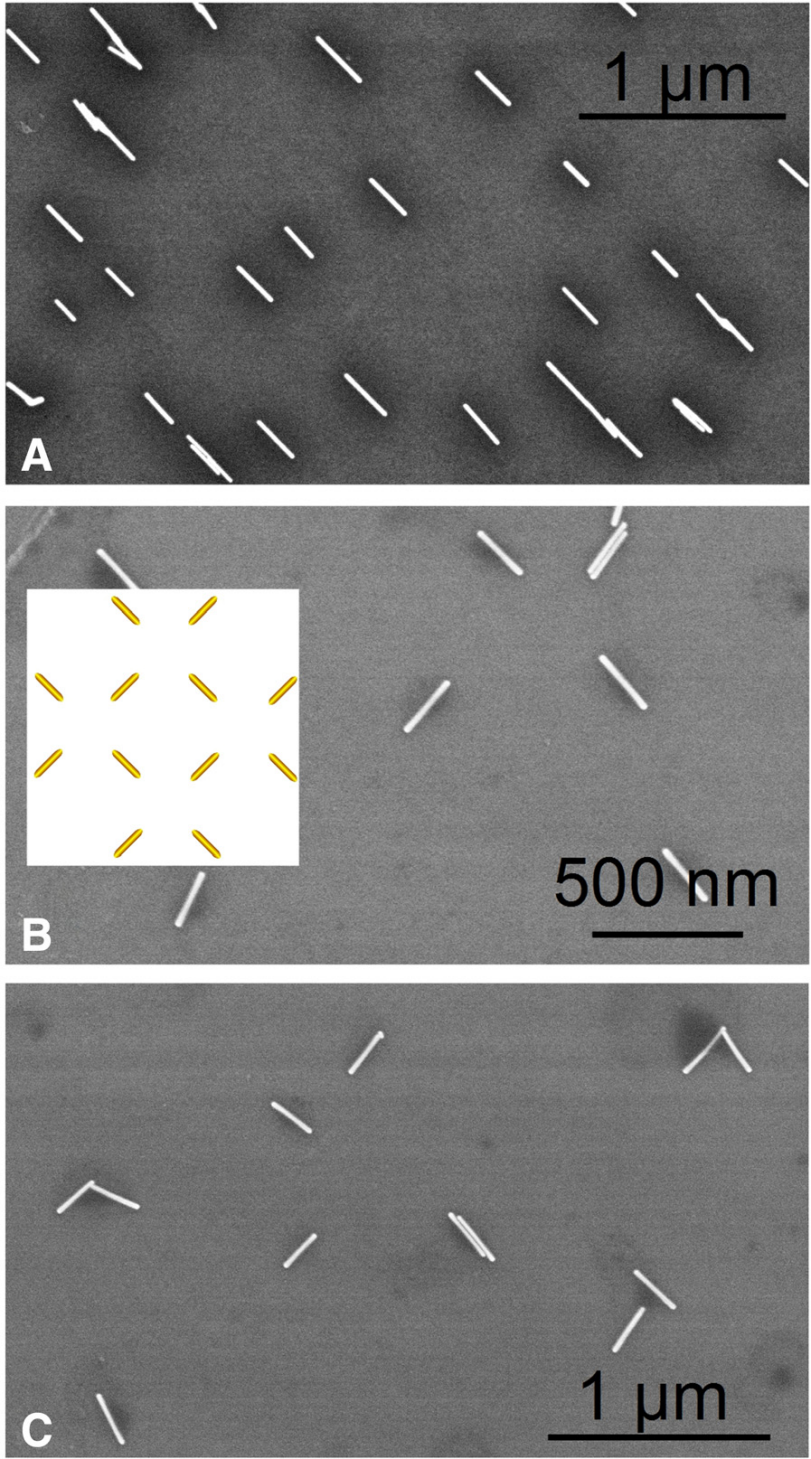
**AuNR arrays with network structures were generated using double assembling.** The linear array was rotated by 90° before making the second assembling. **(A)** The linear array generated from the first assembling. **(B, C)** Network arrays generated after the second assembling.

Normal capillary force was demonstrated to be useful for patterning nanomaterials including spherical nanoparticles [8,23]. Our control experiments show, however, that normal capillary force failed to drive high-AR (ca. 10 to 22 in this work) AuNRs into the nanopores, and arrays of AuNRs could therefore not be obtained. It is reasonable to assume that driving a higher-AR AuNR needs much higher force than driving a spherical gold nanoparticle or a lower-AR AuNR with a comparable diameter. This is due to the fact that a higher-AR AuNR is normally much heavier than a lower-AR AuNR or a spherical nanoparticle. Vacuum created by a simple syringe in the present work provides an enhanced capillary force, making the assembly of high-AR AuNRs successful and efficient.Variants of linear AuNR arrays could be obtained by choosing diverse templates with different dimensions or/and relief structures or by multiple linear assemblies with different orientations on a substrate. Shown in Figure 5 is a proof-of-concept example demonstrating the feasibility to form a two-dimensional AuNR array. After a linear AuNR array (Figure 5A) was produced using the patterning procedure described in Figure 1, we rotated the patterned substrate 90° in its plane and then placed another linear array of AuNRs by carrying out the same patterning procedure. The resultant array displays a two-dimensional network structure (Figure 5B,C), as illustrated in Figure 1B-h.

## Conclusions

We have developed a simple, inexpensive, and efficient procedure that is based on a vacuum-enhanced capillary force for assembling anisotropic AuNRs into AuNR arrays. The AuNR arrays generated are not only single-rod resolved but also highly aligned and ordered. The created vacuum enables fast alignment of high-AR AuNRs on a large scale. Further work is needed to investigate the impact of various parameters on assemblies and to refine the process of forming two-dimensional AuNR nanoarrays as demonstrated in the proof-of-concept example. The approach presented here could be extended to assemble other anisotropic nanostructures including other nanorods/nanowires, carbon nanotubes, and DNA, and the nanoarrays fabricated could find potential applications in nanoelectronics, nanoplasmonics [5,6], SERS sensing [6], and chemical detection [24].

## Competing interests

The authors declare that they have no competing interests.

## Authors' contributions

JW synthesized, assembled, and characterized the gold nanorods and participated in the interpretation of experimental results and in the manuscript drafting. ZW conceived, designed, and guided the research and drafted the manuscript. ML, BT, PX, LM, ZH, and YZ provided assistance in the experiments and discussed the data. All authors read and approved the final manuscript.

## Supplementary Material

Additional file 1 **Supporting information.** Synthesis, separation, and purification of high-aspect-ratio AuNRs; the modification of the silicon surface; DVD templates and the fabrication of PDMS stamps; and additional SEM images of AuNRs both randomly distributed and highly aligned on large-area substrates. Click here for file
