# Supplementary material for: Assembling single gold nanorods into large-scale highly aligned nanoarrays via vacuum-enhanced capillarity
Source: Nanoscale Res Lett. 2014 Oct 7;9(1):556. doi: 10.1186/1556-276X-9-556 (PMC4194060; doi:10.1186/1556-276X-9-556)
Supplement: Additional file 1 — Supporting information. Synthesis, separation, and purification of high-aspect-ratio AuNRs; the modification of the silicon surface; DVD templates and the fabrication of PDMS stamps; and additional SEM images of AuNRs both randomly distributed and highly aligned on large-area substrates. [file 1556-276X-9-556-S1.pdf]

Supporting Information

Assembling Single Gold Nanorods into Large-Scale Highly Aligned Nanoarrays via Vacuum-Enhanced Capillarity

Jiaojiao Wang[1,2]
Email: wangjiaojiao@ihep.ac.cn

Min Li[2]
Email: limin@ihep.ac.cn

Bochong Tang[2]
Email: tangbochong@ihep.ac.cn

Peng Xie[2]
Email: xiep@ihep.ac.cn

Lei Ma[2]
Email: malei@ihep.ac.cn

Zhongbo Hu[1]
Email: huzq@gucas.ac.cn

Yuliang Zhao[2]
Email: zhaoyuliang@ihep.ac.cn

Zhongqing Wei[2*]
[*] Corresponding author
Email: zqwei7@yahoo.com

[1] College of Materials Science and Opto-electronic Technology, University of Chinese Academy of Sciences, Yuquan Rd. 19B, Beijing 100049, China
[2] CAS Key Laboratory for Biomedical Effects of Nanomaterials and Nanosafety, Institute of High Energy Physics, Chinese Academy of Sciences, Yuquan Rd. 19A, Beijing 100049, China

1. DVD templates used to fabricate poly(dimethylsiloxane) (PDMS) stamps

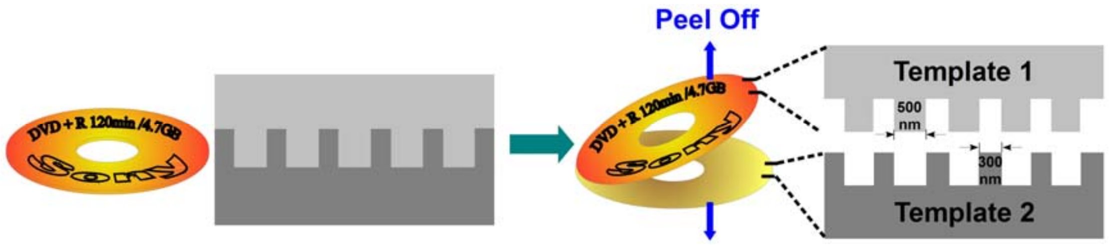

Figure S1. Two types of templates, Template 1 and Template 2, were obtained by peeling off a Sony DVD (Sony Company). The two templates are structurally complementary.

2. The fabrication of PDMS stamps

Normal PDMS stamps were fabricated by casting a 10:1 ratio (v:v) mixture of Sylgard 184 elastomer and Sylgard 184 elastomer curing agent (Dow Corning) on two type of DVD templates (Template 1 and Template 2) having complementary structures. The polymers/templates were then placed in an oven (60 °C, 2 h). After cooling to room temperature, the stamps were carefully peeled from the templates, followed by rinsing with ethanol and drying with nitrogen. The relatively soft PDMS stamps were fabricated using the procedure described above except that the ratio was adjusted to be 15:1.

3. AFM and SEM images of normal PDMS stamps fabricated from Template 2

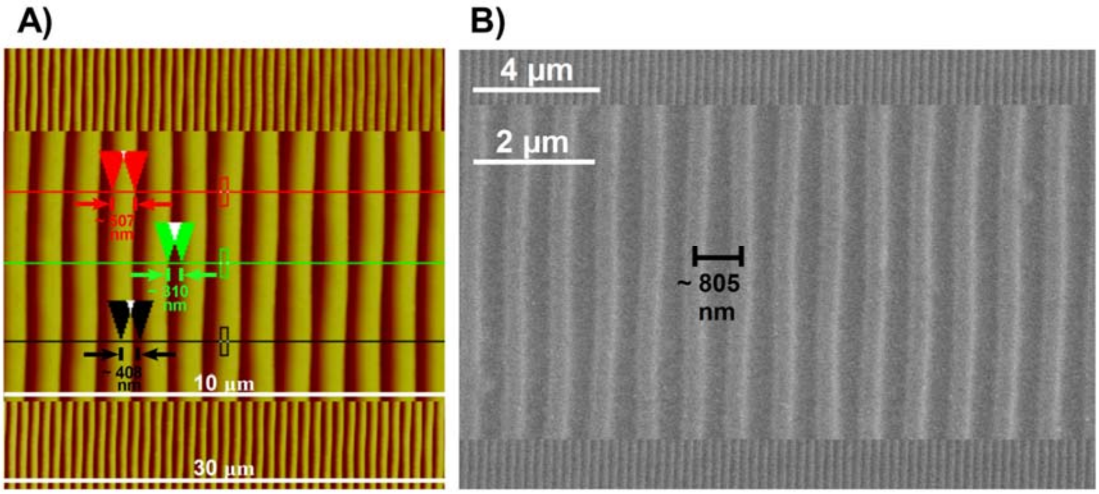

Figure S2. A) Low and high resolution AFM images of a normal PDMS stamp fabricated from Template 2. The width (FWHM) of the raised periodic stripes, generated from the grooves of Template 2, is ca. 507 nm. The width (green) of the grooves of the stamp is ca. 310 nm. The width (black) of half a period of the periodic structure of the stamp is ca. 408 nm. All these data are consistent well with the corresponding sizes of Template 2 and the experimental result shown in Figure 3. **B)** Low and high resolution SEM images of a normal PDMS stamp fabricated from Template 2. The width (black) of a period in this periodic structure, ca. 805 nm, is consistently about twice as much as that of half a period shown in the left-hand AFM image.

4. Synthesis, separation, and purification of high aspect ratio gold nanorods (AuNRs)

1) Preparation of 3-5 nm gold seed solution[1]

The gold seed solution was prepared by adding, while vigorously stirring, 0.6 ml of ice-cold 0.1 M $NaBH_4$ to 20 ml of mixture containing 2.5×10^{-4} M $HAuCl_4$ and 2.5×10^{-4} M tri-sodium citrate. The solution turned purple immediately, indicating the formation of 3-5 nm diameter gold seeds.

2) Synthesis of high aspect ratio AuNRs[1,2]

Three conical flasks were labeled A, B, C, respectively. The growth solution (consisting of 2.5×10^{-4} M $HAuCl_4$ and 0.1 M CTAB) in the amount of 9 ml, 9 ml, and 90 ml was placed into flasks A, B, C, respectively. Then, 0.05 ml, 0.05 ml, and 0.5 ml of 0.1 M aqueous ascorbic acid solution was added into flasks A, B, C, respectively. All three flasks were shaken and the solutions became colorless. After that, 1 ml of solution A was transferred to B after 5 seconds of adding 1 ml seed solution to A. All of the content of the resulting solution B was then transferred into flask C after 10 seconds of adding 1 ml A to B. The flask C was then left undisturbed for 14 hr at 34 ℃. High aspect ratio AuNRs along with faceted 2D platelets precipitate from the solution and deposit on the bottom and the wall of the flask. After carefully removing purple-color supernatant (mostly spherical nanoparticles), the deposit on the wall of the flask as well as on the bottom were carefully rinsed with 5 ml tri-distilled water. The resultant suspension was sonicated (180 W) for ca. 5 s so as to dissolve the deposit in water completely, followed by centrifugation for 5 min at 5000 rpm. The precipitate generated is a mixture of high aspect ratio of AuNRs and 2D platelets. See **panel A** in **Figure S3.**

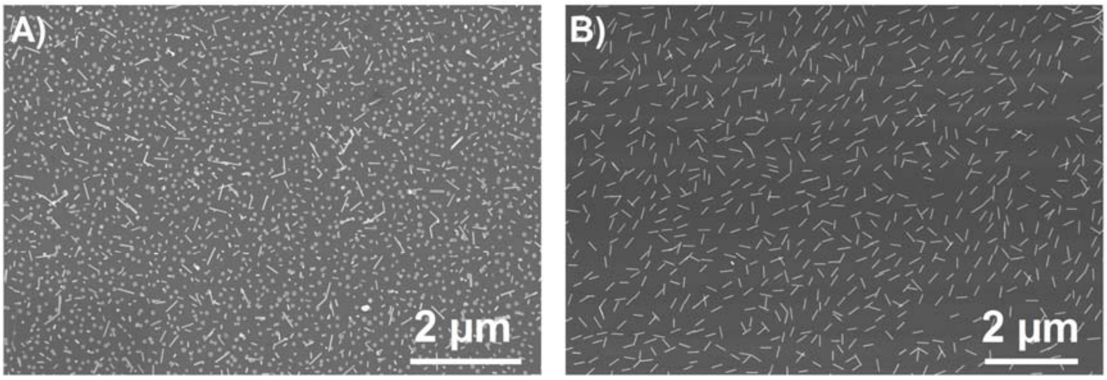

Figure S3. A) SEM image of mixture of faceted platelets and rods after the removal of small spherical particles; **B)** SEM image of AuNRs precipitated from the solution of gold nanodisks and AuNRs after the mixture shown in panel A was treated with Au (III)/CTAB complex.

3) Separation and purification of high aspect ratio AuNRs[2]

Pure AuNRs were separated from the platelets following the following procedure. First, an oxidizing Au(III)/CTAB complex was prepared by dissolving 364 mg of

CTAB and 1.97 mg of $HAuCl_4 \cdot 3H_2O$ in 10 mL of tri-distilled water. Then, the precipitate mixture was dissolved in 2 ml of a mixed solution containing 0.5 M CTAB and 0.5 ml of oxidizing Au (III)/CTAB complex, followed by stirring and leaving undisturbed for 14 hr. As a result, pure AuNRs precipitated while platelets dissolved in the liquid phase. Thus, pure high aspect ratio AuNRs was eventually obtained by removing the green-blue supernatant of dissolved platelets. See **panel B** in **Figure S3.**

5. The modification of silicon surface

The silicon surface was modified following the procedure described previously.[3] Briefly, the Si substrate was first treated with piranha solution (H_2SO_4/H_2O_2, volume ratio 3:1) (**Caution**: piranha solution is highly explosive and should be handled with great care) at 85 °C, followed by rinsing with water and drying with nitrogen gas. The dried substrates were then modified by heating for 30 min in a solution containing 10 ml of 2-propanol, 100 μ L of (3-mercaptopropyl)triethoxysilane (MPTES, Alfa Aesar), and 5 to 6 drops of triple-distilled water. The modified silicon surface was finally rinsed with 2-propanol and dried with nitrogen gas.

6. Figure S4. Additional SEM images for high aspect ratio AuNRs randomly distributed on large-area silicon substrates

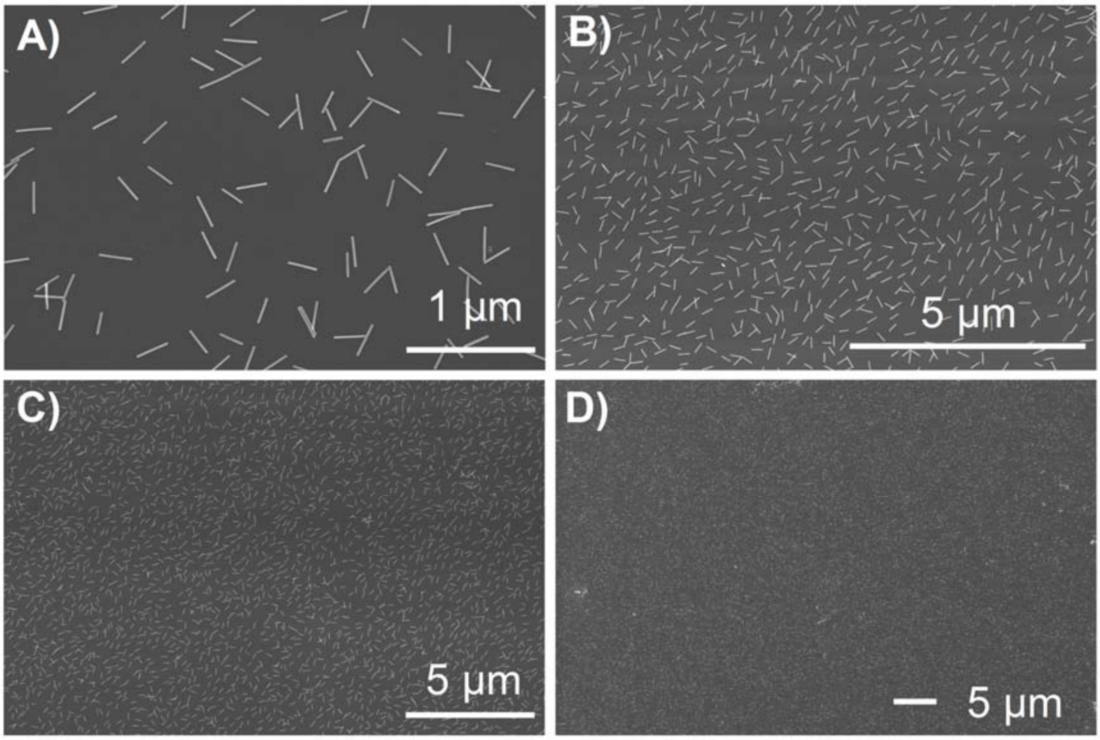

7. **Figure S5. Additional SEM images for high aspect ratio AuNRs highly aligned on large-area silicon substrates**

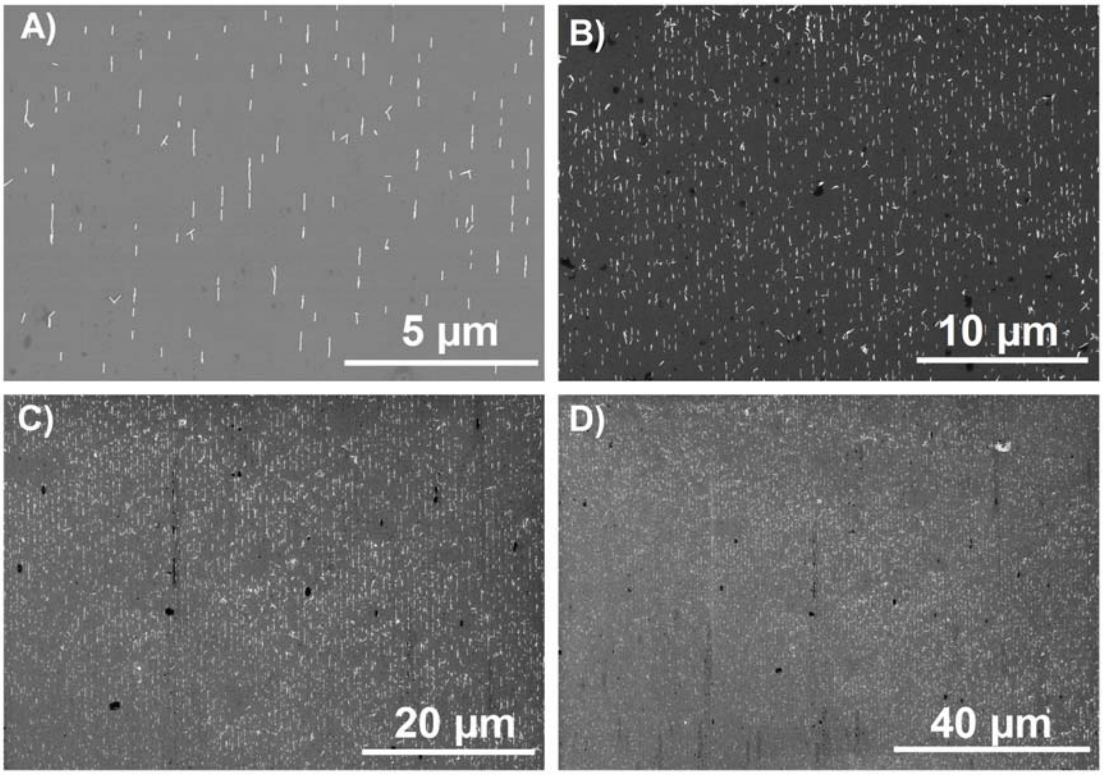

8. Figure S6. Additional SEM image for aligned bundles of AuNRs on a silicon substrate

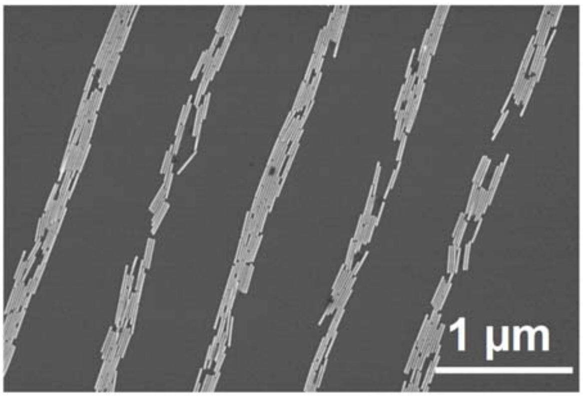

References

1. Jana NR, Gearheart L, Murphy CJ: **Wet Chemical Synthesis of High Aspect Ratio Cylindrical Gold Nanorods.** *J Phys Chem B 2001,* **105**: 4065-4067.

2. Khanal BP, Zubarev ER: **Purification of High Aspect Ratio Gold Nanorods: Complete Removal of Platelets.** *J Am Chem Soc 2008,* **130:** 12634-12635.

3. Wei Z, Mieszawska A, Zamborini FP: **Synthesis and Manipulation of High Aspect Ratio Gold Nanorods Grown Directly on Surfaces.** *Langmuir 2004*, **20:** 4322-4326.
